# Relationship of Gonorrhœa to Diseases of the Female Pelvic Organs

**Published:** 1895-05

**Authors:** Russell Caffery

**Affiliations:** San Antonio, Texas


For Texas Medical Journal.

### RELATIONSHIP OF GONORRHEA TO DISEASES OF THE FEMALE PELVIC ORGANS.

BY RUSSELL CAFFERY, M. D., SAN ANTONIO, TEXAS.

ATTENTION is continually being called to the fact that chapters of modern, as well as ancient text-books devoted to the subject of the importance of gonorrhea as an etiological factor in the disease of the female pelvic organs, are characterized by a brevity and lack of harmony equally misleading and confusing.

The reason for this is not obvious unless it be in the fact that different observers, employing widely varying methods for diagnostic purposes, necessarily arrive at different, if not faulty conclusions.

It is true, an accurate knowledge of what the gonococcus of Neisser will and will not do, when liberated in the genital tract of a woman free from already existing disease, is denied even to the most careful observers, yet an approximate idea is obtained by the intelligent study of the anato-pathological lesions found during repeated operations upon cases of pelvic disease of unquestioned gonorrheal origin.

There should be an increasing demand for a more definite knowledge of this subject, and very properly, too, for the magnitude of the question can not be overestimated when its effect upon the health, happiness and general welfare of a vast number of the human family is taken into consideration.

As a rule, the general practitioner finds little time left him for investigation into the etiology of pelvic disease, therefore feels that he must depend largely upon the opinion of some one of his specialist brethren for guidance. This is unfortunate, and it would seem eminently proper for physicians, when called upon for diagnosis in an acute inflammatory condition of the genital tract, to disregard all theories and isms, and proceed with the profound consciousness of the importance of early diagnosis, to bring to bear all modern and scientific means for diagnostic purposes, such as a clear previous history, existing lesions, physical and microscopical character of utero-vaginal discharge, would afford us. (It is not believed that the actual transmission of the contents of a diseased uterus and vagina, by way of the lymphatics, is demonstrable upon the living subject, therefore fails of diagnostic value.)

The necessity for early diagnosis, followed by prompt interference, will be realized when physicians generally come to understand that the infective principle of gonorrhea is the most widely diffusible, and is followed by the most rapidly destructive pathological process known as being common to the genital tract.

The ancient ipse dixit that a specific urethral discharge in the male becomes innocuous when it reaches the subacute stage, still finds favor with a few; it is misleading, and will certainly result disastrously if allowed to prevail when determining the cause of acute disease in the female pelvis.

Differential diagnosis sometimes has for a basis the social rank of individuals, which can not be too severely condemned; for what is diagnosed as gonorrhea in the courtesan should not be called catarrh of the womb when found invading the home precincts of society's pace makers.

These remarks, it might be said, were occasioned by my observations in ten cases of tubo-ovarian disease of undoubted gonorrheal origin, under treatment recently. Out of the entire number, I was familiar with the condition of pelvic contents of three of them previous to infection; the other seven came under observation after the disease had progressed to encysted pus tubes and abscessed ovaries. The necrotic process was, without exception, more extensive on the left side, and all complained of those symptoms that indicated localized pelvic peritonitis, with its concomitant ills. Nine of these cases submitted to abdominal section, with two deaths resulting; the tenth waived all operative interference.

By this rough analysis of the history and pathological condition presented, it is believed some very interesting lines of thought are suggested, lines upon which we must all travel before we are prepared to stagger around under the burden of a surgical diathesis. So, then, the not very flattering record of two deaths out of nine celiotomies is hereby presented, but as statistics are only valuable to the surgeon when they are highly classified, therefore, in grouping these cases, the aim has been to eliminate everything not belonging to this distinct class of disease. Finally, let it be said, that no effort has been made to ascribe to gonorrhea any undue etiological importance in diseases of the female pelvic organs, but instead, a plea is meant to be entered for a more thorough and systematic effort, on the part of physicians, to determine the specific or non-specific character of utero-vaginal discharges when called upon to care for women suffering from acute vagino-endometritis.

